# Mounting Evidence for an Expanded Host Range of Influenza B Viruses

**DOI:** 10.3390/v17121528

**Published:** 2025-11-21

**Authors:** Marios Koutsakos, Rhys H. Parry, Michelle Wille

**Affiliations:** 1 Department of Microbiology and Immunology, University of Melbourne at the Peter Doherty Institute for Infection and Immunity, Melbourne, VIC 3000, Australia; 2 School of Chemistry and Molecular Biosciences, The University of Queensland, Brisbane, QLD 4067, Australia; r.parry@uq.edu.au; 3 WHO Collaborating Centre for Reference and Research on Influenza, at the Peter Doherty Institute for Infection and Immunity, Melbourne, VIC 3000, Australia

**Keywords:** influenza B, virus ecology, animal reservoirs

## Abstract

Influenza B viruses (IBV) belong to the family of *Orthomyxoviridae* and circulate annually in humans causing respiratory illness. Although they are considered an exclusively human pathogen, there is evidence of IBV infections in animals, including wildlife, companion animals and livestock. In addition, metagenomic studies have identified novel orthomyxoviruses in amphibians and fish that appear related to IBV, suggesting influenza viruses, including IBV, have been associated with vertebrates across their evolutionary history. In this review, we summarise our current knowledge of potential IBV and IBV-like infections in animals. These collectively suggest that the ecology of IBV extends beyond humans and warrants further investigations.

## 1. Introduction

The family of *Orthomyxoviridae* includes four ratified viruses: influenza A, B, C, and D viruses, which are well-established agents of respiratory illness in humans and animals [1,2,3]. Of these, influenza A viruses (Genus: *Alphainfluenzavirus*, IAV) are of high pandemic risk owing to their role in previous human pandemics, the large diversity found in various animals, and their ability to jump into humans and cause severe or fatal infections [1,2,4]. Influenza D viruses (Genus: *Deltainfluenzavirus*, IDV) are found in cattle, while influenza B viruses (Genus: *Betainfluenzavirus*, IBVs) and influenza C viruses (Genus: *Gammainfluenzavirus*, ICV) circulate in humans but have no known established animal reservoir. Without an animal reservoir through which to harbour virus diversity, IBVs and ICVs are not considered to pose a pandemic threat and have therefore been considerably overlooked [5,6]. Despite this, IBV causes a considerable clinical and economic burden in the human population [7,8,9]. While two antigenically distinct IBV lineages (B/Yamagata and B/Victoria) had been co-circulating since the late 1980s, the B/Yamagata lineage went extinct in 2020, most likely as a result of non-pharmaceutical interventions implemented during the COVID-19 pandemic [10,11]. The extinction of one IBV lineage, combined with the absence of an animal reservoir, makes the eradication of IBV from humans, at least theoretically, plausible [12]. For virus eradiation to be considered or implemented, we need a clear understanding of system-level epidemiology, ecology, and evolution. At present, IBV is considered a human-restricted virus, but sporadic evidence from animals challenges this notion. Indeed, multiple lines of evidence suggest that IBV has considerable potential for reverse zoonosis [13,14,15,16,17,18] and a poorly understood presence in pinnipeds [19,20,21,22,23,24]. Recent metagenomics has also demonstrated the existence of influenza B-like viruses in basal vertebrates and amphibians [25,26], suggesting co-evolution with vertebrate hosts, which indicates that a diversity of IBV or IBV-like viruses may exist in higher vertebrates. These observations exemplify our incomplete understanding of IBV ecology. In this review, we summarise our understanding of IBV host range and highlight important knowledge gaps that need to be addressed to fully appreciate the existence of any non-human IBV and IBV-like viruses, as well as the threats they pose to humans and animals, if any.

## 2. Experimental Models of IBV Infection to Inform Transmission and Host Species Restriction

Experimental infection models are crucial for investigating IBV host range, pathogenesis, and transmission potential, helping to distinguish potential reservoirs from dead-end hosts. IBV can infect a variety of mammalian species, often without adaptation. This observation indicates that IBV is not inherently restricted to humans as a host, but rather that a diversity of mammalian species is susceptible to the virus if exposed. In addition, these models provide insights that may help interpret whether natural infections represent sustained circulation or incidental spillover events.

Ferrets (*Mustela putorius furo*) are considered the gold standard for human influenza virus research, largely because their respiratory tract physiology and sialic acid receptor distribution closely mimics that of humans [27]. Ferrets are susceptible to unadapted IBV, though disease severity varies by strain, with B/Brisbane/60/2008 causing the most significant clinical illness and efficient lower respiratory tract infection [28]. Transmission of unadapted IBV in ferrets has been variable, with some studies reporting limited [29] or efficient contact transmission [30,31,32] and both efficient and limited airborne transmission [29,31,32,33], noting that different isolates were used among studies. Mice (*Mus musculus*) can also support the replication of unadapted IBV strains [33,34,35,36], although laboratory adaptation through serial viral passage can enhance pathogenesis [29,37]. Guinea pigs (*Cavia porcellus*) have emerged as a valuable model for studying IBV transmission. Unadapted human IBV replicates efficiently in the upper respiratory tract, facilitating efficient spread via both direct contact and aerosols [38]. Other model animals which are susceptible to unadapted IBV include tree shrews (*Tupaia belangeri*) and cotton rats (*Sigmodon hispidus*). In tree shrews, infection leads to clear pathogenesis markers like weight loss, viral replication, and cytokine responses despite mild upper respiratory signs [39]. Cotton rats support robust replication of various unadapted IBV strains in both the nose and lungs [40]. Notably, while demonstrating susceptibility and pathogenesis, transmission between individuals was not assessed in these specific key studies for either tree shrews or cotton rats, leaving their capacity for onward spread an open question. Domestic pigs (*Sus scrofa domesticus*) can also be experimentally infected with both lineages of unadapted IBV, developing clinical signs and lung lesions. Crucially, demonstrated pig-to-pig transmission via direct contact for at least one strain (B/Brisbane/60/2008) highlights their potential as IBV hosts, though whether they may act as a significant natural reservoir remains unclear [14]. Syrian hamsters (*Mesocricetus auratus*) are susceptible to unadapted IBV, supporting good viral replication, particularly in the lungs, often without overt clinical symptoms, and are not capable of airborne transmission of IBV [41]. As most of these studies focus on viral replication and transmission, it would be worthwhile to also explore immune responses in different animal models.

While several mammalian species can be experimentally infected with unadapted IBV, as described above, only those demonstrating efficient onward transmission have characteristics suggesting they could play a role in IBV ecology outside of humans, potentially sustaining circulation. It is also worth investigating alternative routes of transmission for IBV, including through water or contaminated food sources. Species that readily get infected but either fail to transmit in experiments or haven’t been adequately tested for transmission are more likely to represent epidemiological dead-ends if infected naturally, limiting their significance in IBV’s broader ecology. Therefore, evaluating the capacity for sustained transmission is critical when assessing if a species could contribute to the natural ecology of IBV as a reservoir, rather than just being an incidental spillover host.

## 3. Evidence of IBV Infections in Animals

IBV infections in animals have been reported as early as the 1960s (Table 1), including in domestic animals (horses, pigs, goats, sheep), companion animals (dogs, guinea pigs), game animals (bamboo rats [*Rhizomys* sp.]), and non-human primates (orangutans, gorillas, chimpanzees) [13,14,15,16,17,18,42,43,44,45,46]. Typically, such infections have been inferred from retrospective serosurveys and not from investigations of active disease outbreaks, and the infecting virus is typically inferred based on the specificity of the antibodies detected (for instance, haemagglutination inhibition of human IBV isolates). As a result, a substantial limitation is that while many of these animals are IBV sero-positive, it is unknown exactly what they were infected with. Pigs are an exception, as virus sequence data is available. Specifically, Ran et al. (2015) [14] and Tsai et al. (2018) [15] both found evidence of active IBV infection in swine (in the USA and Taiwan, respectively), with limited sequencing demonstrating the IBV from swine was >99% similar to contemporary human IBVs. There are additional sequences in GenBank of IBV in pigs (e.g., MG692771-78), again, consistent with reverse zoonosis of IBV. There is also genomic evidence of reverse zoonosis of IBV in bamboo rats (as game animals), with partial recovery of all segments from metagenomic data [45]. Given evidence for reverse zoonosis in pigs and bamboo rats and that most serological detections are against human IBV isolates and in species with direct contact with humans, it is widely assumed that serological detections of IBV from companion animals, livestock, and non-human primates (Table 1) are caused by reverse zoonosis events from humans; however, this is largely unconfirmed.

In addition to animals in direct contact with humans, there is considerable evidence for IBV infection in wild pinnipeds (i.e., seals and sea lions). IBV was first reported in seals in the Netherlands in 1999, where twelve harbour seals (*Phoca viulina*) were admitted to an animal hospital with respiratory disease, and an IBV with high similarity (NS segment 99% identical to human IBV NS) to human strains was subsequently isolated. Serology demonstrated further evidence of IBV antibodies in both harbour seals and grey seals (*Halichoreus grypus*) in the Netherlands in samples collected in 1995 and later [20]. These findings suggested a reverse zoonosis event of human IBV into pinnipeds in the mid-1990s. Further serological evidence from the Netherlands indicated that this was not an isolated jump into seals followed by extinction in the population, but rather that IBV continued to circulate among pinnipeds of that region until at least 2012. Critically, some of the samples were from juvenile animals (<1 year) collected in 2009 (i.e., 10 years after the initial detection), which is suggestive of continued transmission [19]. The detection of a virus with a high homology to humans, as well as the detection of antibodies by a hemagglutination assay using human IBV isolates, supports the idea that infections of pinnipeds in the Netherlands may have been a result of reverse zoonosis. However, IBV seropositivity has been subsequently detected in additional pinniped species (hooded seal *Cystophora cristata*, grey seal, harbour seal, harp seal *Pagophilus groenlandica*, South American fur seal *Arctocephalus australis*, Caspian seal *Phoca capsica*) in Canada, Uruguay, and the Caspian Sea [21,22,23,47]. In addition to serology, there has also been a metagenomic detection of small IBV genomic fragments in tissues from a dead Caspian seal in 2020 [24]. Overall, although there is some serological evidence of IBV infections in pinnipeds across different geographic locations and species, suggesting that pinnipeds are highly susceptible to IBV infection, the role of pinnipeds in IBV ecology is unclear.

**Figure 1:**
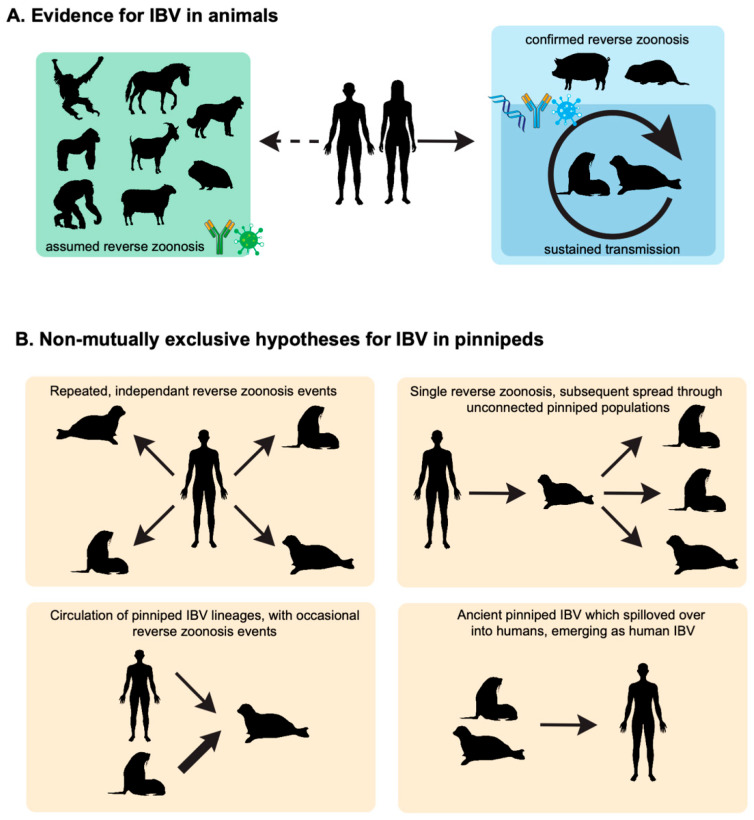
IBV in animals. (**A**) Overview of IBV ecology. IBV circulate in humans with multiple reverse zoonosis events in animals, including wildlife, companion animals, and livestock. Some of these reverse zoonosis events have been confirmed by the molecular detection of IBV genomes in pigs and pinnipeds, while most have been inferred based on various serological methods. The sustained transmission of IBV has also been suggested based on the widespread seropositivity of pinnipeds to IBV. (**B**) Speculative hypothesis about the role of pinnipeds in IBV ecology. Silhouettes from phylopic.org (https://www.phylopic.org/, accessed on 17 October 2025) and bioicons.com (https://bioicons.com/, accessed on 17 October 2025).

The scant evidence from pinnipeds raises four potential hypotheses about the role of pinnipeds in IBV in ecology (Figure 1), which are not mutually exclusive and remain largely speculative: (1) multiple reverse zoonosis events from humans into pinnipeds occur, repeatedly introducing IBV to diverse pinniped populations, (2) a single reverse zoonosis event has occurred, and IBV has somehow spread across unconnected pinniped populations, (3) pinnipeds have one or more independent lineages of non-human IBV (with potential human IBV spill-overs into pinnipeds, which may result in lineage replacement or co-circulation), and (4) pinnipeds represent the ancestral source from which human IBV originally emerged. Hypotheses (1) and (2) would require extensive interactions between humans and pinnipeds or extensive spread of IBV between geographically separated pinniped populations. However, the likelihood of such events is difficult to ascertain. While hypotheses (3) and (4) may seem more plausible, it must be stressed that these hypotheses are entirely speculative and currently lack supporting data. Notably, there is no genomic or virological evidence to date supporting the existence of distinct non-human IBV lineages in pinnipeds. These significant uncertainties highlight the need to further dissect the role of pinnipeds in IBV ecology.

The presence of IBV in animals, temporarily or as a reservoir, has potential implications beyond a fundamental understanding of virus ecology. Firstly, even over a short period of circulation in animals after reverse zoonosis, other viruses like IAV and SARS-CoV-2 have undergone diversification and subsequently reinfected humans [48,49,50,51]. Secondly, even in the absence of diversification, the maintenance of an ancestral human virus lineage in an animal population might become a risk for humans as population immunity to that lineage wanes. Such an ‘antigenic archive’ of human IAV strains was reported in swine populations [52]. Consequently, the presence of these old lineages (which may be extinct in humans) in animals raises the risk of zoonotic transmission and the re-emergence of past influenza variants. Whether seals play a similar role for past human IBV diversity is unknown, but it is worth considering given the putative long-term transmission in seals in the Netherlands. Finally, it is also pertinent to understand the clinical impact of IBV infection in animals as this may aid in the differential diagnosis of IBV in animals. As most studies on IBV in animals have been serological and thus are post-infection studies, it is not possible to ascertain the disease status of antibody-positive animals. The two studies undertaken on pinnipeds in the Netherlands both reported juvenile seals in respiratory distress, but did not ascertain whether IBV was the causative agent and could not rule out the role of other coinfections [20]. Experimental infection of pigs resulted in influenza-like symptoms and lung lesions [14], but it is not known if natural infection has the same outcomes.

Collectively, these observations from animals indicate that IBV can infect a variety of mammalian species in different contexts. Additional studies of IBV in animals, including longitudinal monitoring across diverse regions, would prove informative. Current evidence is sparse, and therefore it remains unclear whether IBV has the potential for sustained transmission and spread in animal populations, and given all sequenced genomes thus far suggest reverse zoonosis, there is currently no clear evidence of an established animal reservoir.

## 4. IBV-like Viruses from Fish and Amphibians

**Figure 2:**
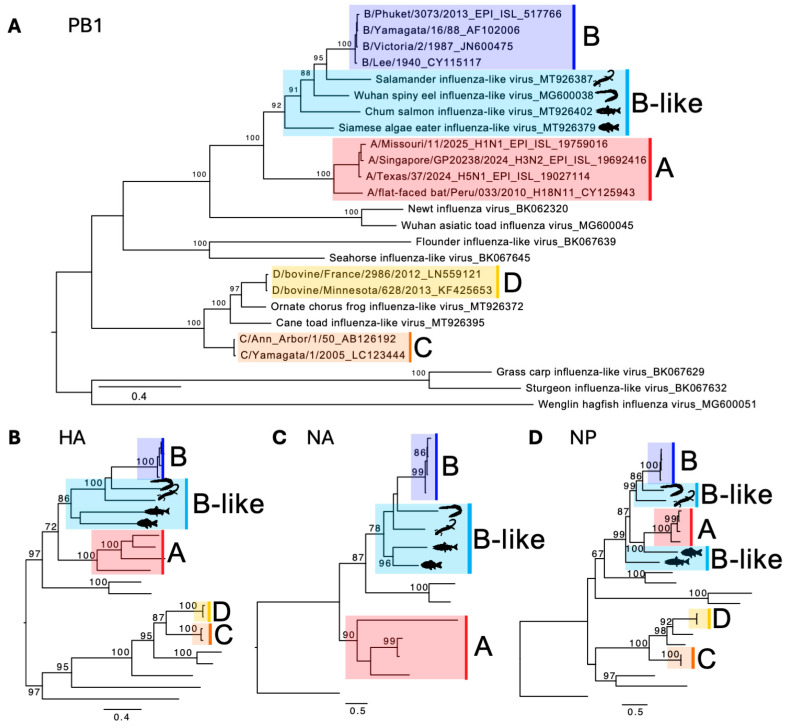
Phylogenetic relationships of vertebrate influenza-like viruses. Maximum likelihood trees of (**A**) the PB1 segment encoding the RNA-dependent RNA polymerase, (**B**) the HA segment, (**C**) the NA segment, and (**D**) the NP segment of various influenza and influenza-like viruses. The four influenza subtypes (A, B, C, D) and B-like viruses are color-coded and labelled. Amino acid alignments were constructed using MAFFT and the E-ins-L algorithm, and trees were constructed using iq-tree2 with the best-fit substitution model and 1000 ultrafast bootstraps. Silhouettes from phylopic.org (https://www.phylopic.org/, accessed on 17 October 2025). Wenling-hagfish influenza virus is the outgroup for all trees. Scale bar is the number of amino acid substitutions per site.

Traditionally, virus discovery has been driven by clinical or epizootic investigations, for instance, Middle East respiratory syndrome coronavirus (MERS-CoV) was first identified after clusters of severe pneumonia in Saudi Arabia in 2012 [53]. However, the advent of metagenomics has dramatically expanded our understanding of viruses in asymptomatic or apparently healthy animals, reshaping our view of viral evolution and host associations [26,54,55]. Indeed, through metagenomics, four novel, coding-complete influenza-like genomes from amphibians and fish that share significant similarity with IBV have been discovered [25,26]. While they are structurally and genetically related to IBV, they are sufficiently divergent and are thus referred to as “IBV-like” viruses. They include salamander influenza-like virus (SILV) genomes identified in RNA libraries from various tissues of Mexican walking fish (*Ambystoma mexicanum*) and plateau tiger salamander (*Ambystoma velasci*); Wuhan spiny eel influenza-like virus (WSEIV) identified in the gill tissues of lesser spiny eels (*Macrognathus aculeatus*); chum salmon influenza-like virus (CSILV) genome identified in an RNA library of gill tissue from chum salmon (*Oncorhynchus keta*); and Siamese algae-eater influenza-like virus (SAEILV) identified in an RNA library from gill tissues from Siamese algae-eater (*Gyrinocheilus aymonieri*). These viruses are basal to IBV in the PB1 phylogeny, as well as in the HA and NA phylogenies (Figure 2) (although they do not form a monophyletic clade), and thus play a crucial role in understanding the diversity and evolutionary history of IBV, as well as of orthomyxoviruses in general.

**Figure 3:**
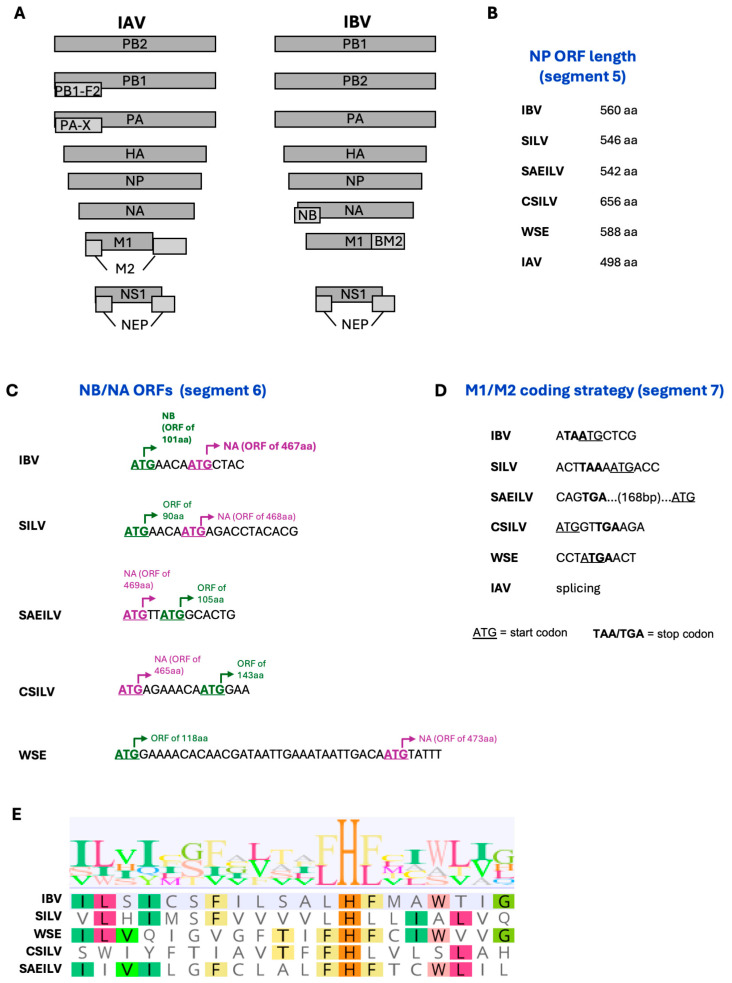
Genomic characteristics of influenza B-like viruses. (**A**) Genome architecture of influenza A and B viruses. (**B**) Amino acid length of NP in IBV and IBV-like viruses. (**C**) Open reading frames of segment 6 of IBV and IBV-like viruses. (**D**) Stop and start codons of M1 and M2, respectively, on segment 7 of IBV and IBV-like viruses. (**E**) Amino acid alignment of the M2 ion channel residues 7–26 (B/Lee/40 numbering).

Their classification as IBV-like viruses is further supported by their similarity to IBV in terms of genome architecture (Figure 3A). The overall genome architecture of IBV has at least three particularly distinctive features compared to IAV [6]: (i) an extended NP open reading frame (ORF) on segment 5 (>540 aa vs. <500 aa for IAV), (ii) the NB ORF (absent in IAV) on segment 6 overlapping with the NA ORF, and (iii) the use of a stop-start pentanucleotide sequence for the expression of the BM2 protein (produced by alternative splicing for IAV M2). All four IBV-like viruses have an extended NP ORF on segment 5 (Figure 3B). In addition, segment 6 sequences from all four IBV-like viruses have a second putative ORF of 90–143 aa that has similarity to the human IBV NB ORF and overlaps with the NA ORF (Figure 3C). However, it is worth noting that while in human IBV, in SILV, and in WSEIV the putative NB ORF is upstream of the NA ORF by 4 (IBV and SILV) or 31 (WSEIV) nucleotides, in SAEILV and in CSILV the order is reversed, and the NA ORF is upstream of the putative NB ORF by two (SAEILV) or seven (CSILV) nucleotides. Finally, the putative M2 ORF on segment 7 of these viruses is likely not encoded by alternative splicing, as in IAV, but rather through the use of stop/start codons, although the detailed mechanisms of this seem to vary significantly (Figure 3D). The most similar to human IBV (TAATG pentanucleotide) is SILV, in which the M1 stop codon (TAA) is separated from the M2 start codon (ATG) by one nucleotide. In SAEILV, the M1 stop codon (TGA) is separated from the M2 start codon (ATG) by 168 nucleotides. Meanwhile in CSILV, the putative M2 start codon precedes the M1 stop codon by two nucleotides, while in WSEIV the putative M2 start codon precedes but overlaps with the M1 stop codon in a 4-nucleotide sequence (ATGA). We note that this is based on analysis of putative ORFs with M2 being identified as the ORF containing the HxxxW amino acid motif required for H^+^ conductance by the M2 ion channel (Figure 3E), which is well-conserved across IBV, IAV, and the IBV-like viruses, with the exception of SILV and CSILV, where the W is absent. Nonetheless, these sequence data suggest that the M2 encoding mechanism of IBV-like viruses is dissimilar to that of IAV (splicing) and more similar to that of IBV (use of stop/start codons). Overall, the extended NP ORF, the presence of a putative NB-like ORF, and the expression mechanism of the putative M2 ORF support the idea that these viruses are related to IBV, although considerable differences between IBV-like and IBV still exist and warrant further investigation to understand the functional homology of the IBV-like encoded proteins.

**Figure 4:**
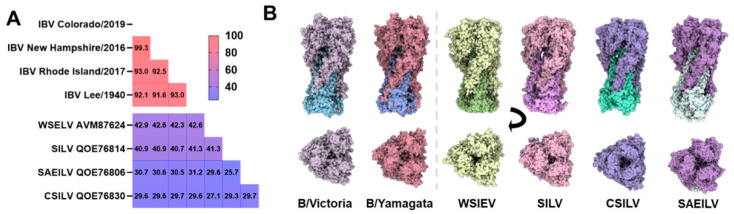
(**A**) Global protein sequence identity and (**B**) structural comparison of IBV and influenza B-like virus hemagglutinins (HA). Structures were superimposed based on their core folds using the matchmaker command in UCSF ChimeraX (v1.11). The upper row shows the side view of the HA trimers, while the lower row shows the apical (top-down) view, obtained by rotating the structures 90 degrees. From left to right, (1) IBV B/Victoria-lineage HA (B/Brisbane/60/2008, PDB: 4FQM) (2) IBV B/Yamagata-lineage HA (B/Yamanashi/166/1998, PDB: 4M44) (3) WSEIV HA (PDB: 9O5W) (4) AlphaFold3 predicted SILV HA (5) AlphaFold3 predicted CSIV HA (6) AlphaFold3 predicted SAEILV HA.

Structural and functional characterisation of the HA and NA glycoproteins from these IBV-like viruses have provided strong evidence that these are indeed *bona fide* homologues of their human IBV counterparts, although certain questions remain unanswered. There is high amino acid similarity in the core polymerase machinery of human IBV and IBV-like viruses from amphibians and fish, with the PB1 protein sharing 67–75% identity with IBV [25]. In contrast, the pairwise amino acid similarity across the HA protein (Figure 4A) is relatively lower (~27–31% between the IBV-like and human IBV). For context, we also note the HA of A/flat-faced bat/Peru/033/2010(H18N11) shares only 36.3% amino acid similarity to A/Singapore/GP20238/2024(H3N2), both of which are considered IAV. Despite this low sequence identity, there is significant structural homology between the HAs of IBV and IBV-like viruses (Figure 4B). The cryo-EM structure of the WSEIV (PDB: 9O5W) [56] aligns remarkably well with resolved IBV HA structures from both major IBV lineages. Structural alignment undertaken using matchmaker on ChimeraX (v1.11) shows a pruned RMSD of just 1.043 Å (over 227 atom pairs) when compared to the B/Victoria-lineage HA (PDB: 4FQM), and an even closer fit of 0.970 Å (over 232 pruned atom pairs) when compared to the B/Yamagata-lineage HA (PDB: 4M44). This demonstrates a highly conserved structural fold, particularly in the core, despite extensive divergence in the amino acid sequence.

Attempts to obtain high-resolution cryo-EM structures for SAEILV, CSILV, and SILV have been unsuccessful. However, low-resolution negative stain EM maps acquired for SAEILV and SILV HA showed that while SILV HA resembles the canonical shape of influenza virus HA, SAEILV HA possesses an open and flexible head domain, making high-resolution determination difficult [56]. To complement the experimental data, in silico AlphaFold3 models for the trimeric HA of SILV (GenBank: QOE76814), SAEILV (GenBank: QOE76806), and CSILV (GenBank: QOE76830), trimers of HA_1_-HA_2_ based on their respective HA_1_ (SILV: 23–360; SAEILV: 17–346; CSILV: 23–354) and HA_2_ (SILV: 361–541; SAEILV: 347–528; CSILV: 355–534) domains were generated. The predicted models were high quality, with strong overall confidence (ipTM/pTM = 0.81 for CSIV, 0.79 for SILV; pTM = 0.72 for SAEILV) and high local confidence (pLDDT > 90) in their globular head domains. While structural comparisons based on predicted models must be approached with caution, we observed that the AlphaFold3 models of CSILV, SAEILV, and SILV all shared a high degree of structural homology with the experimental cryo-EM map of WSEILV (9O5W) (Figure 4B).

The function of the IBV HA is attachment to host cells via sialic acid receptors and membrane fusion in the endosome to release the viral genome in the host cell. Current evidence suggests that the HA proteins of IBV-like viruses likely conform to this function (Table 2). Despite the overall similar structure, the regions surrounding the receptor binding site (RBS) of the HA exhibit less conservation [56]. Consistently, the receptor specificity of the different HAs varies. While human IBV HA can interact with both α2,3-linked sialic acid and α2,6-linked sialic acid, only SAEILV and CSILV HAs can recognise α2,3-linked sialic, with SAELV also recognising sialyated Lewis X moieties. On the other hand, the WSEIV HA recognises monosialic ganglioside 2 (GM2), while SILV does not recognise any of the tested glycans [56,57]. Consistent with these results from glycan microarrays, SAEILV and CSILV HA proteins can agglutinate turkey and chicken RBCs [56,57]. Following receptor engagement, the primary role of the HA is to mediate membrane fusion at the low pH of the endosome. In polykaryon-based assays, human IBV as well as SAEILV and WSEIV HAs exhibit fusogenic potential at various pH levels (4.5–5.9), supporting their ability to mediate membrane fusion [56,57]. While SILV and CSILV HAs do not mediate membrane fusion, this likely reflects the requirement for proteolytic activation of the HA0 precursor by host proteases, which varied between IBV-like HAs. For human IBV, this can be achieved by a variety of type II transmembrane serine proteases (TTSPs) and kallikrein (KLK) enzymes expressed by airway cells [58], as well as by exogenously added N-tosyl-L-phenylalanine chloromethyl ketone (TPCK)–treated trypsin. The SAEILV HA is cleavable by select TTSPs and KLK enzyme and trypsin [56], while the WSEIV HA is not cleavable by any of the tested human TTSP or KLK enzymes but is cleavable by trypsin [57]. In contrast, neither SILV nor CSILV HAs are cleavable by any of the tested human TTSP or KLK enzymes or by trypsin [56]. These differential cleavability patterns explain the observed lack of fusogenic potential for SILV and CSILV HAs, as proteolytic cleavage is required for membrane fusion. More importantly, they indicate substantial differences in the biology of the IBV-like HA proteins and likely cell and host-tropism, all of which warrant further investigation.

In contrast to the HA that mediates attachment to host cells, the NA is responsible for cleavage of sialic acid receptors, primarily to facilitate egress and budding of new virions [59]. Characterisation of the WSEIV and SAELV NAs has provided novel insights into their biology, while the NAs of CSILV and SILV have not been successfully expressed yet. Both WSEIV and SAELV NAs, similar to the human IBV NA, showed temperature-dependent enzymatic activity in an enzyme-linked lectin assay, with the highest activity at 37 °C compared to lower temperatures (33 °C, 20 °C, 4 °C) [56,57]. Structural characterisation of the WSEIV and SAEILV NAs demonstrated significant sequence and structural conservation of the enzymatic active site, as well as the presence of calcium binding sites similar to that of human IBV NAs [56]. However, the remaining surfaces of the IBV-like NAs are less conserved. The conservation of the enzymatic active site, however, appears sufficient for maintaining susceptibility to NA inhibitors like oseltamivir, zanamivir, and peramivir. Indeed, both WSEIV and SAEILV NAs are similarly susceptible to inhibition by all three compounds [56,60]. Overall, the NAs of IBV-like viruses appear to be functional homologues of human IBV NAs.

As the RBS of the HA and the enzymatic active site of the NA are conserved and the majority of the remaining surface of each glycoprotein is not well conserved between IBV and IBV-like viruses, the surface glycoproteins of IBV-like viruses are antigenically distinct from those of human IBV [56,57]. Indeed, these antigens are not cross-recognised by human adult sera. However, the WSEIV HA can be cross-recognised by some murine monoclonal antibodies (mAbs) primarily targeting the HA stem domain and the WSEIV NA can be cross-recognised by the IAV/IBV universal anti-NA mAb 1G01, but not other mAbs. In contrast, the SAEILV, CSILV and SILV HAs and NAs cannot be cross-recognised by any of the tested mAbs, with the exception of the IBV universal anti-NA mAb 2E01, which weakly bound the SAEILV NA. Overall, the HA and NA glycoproteins of IBV-like viruses are antigenically distinct from their human IBV counterparts and humans do not possess cross-protective humoral immunity against them. Future examination of conserved T-cell or B-cell epitopes, particularly those involved in cellular immunity, could reveal potential cross-protection relevant to zoonotic events. While these may suggest susceptibility of the human population to these viruses, IBV-like viruses from fish and amphibians would certainly face strong species barriers as evident from the functional characterisation of the SILV and CSILV HAs. Even in the case of SAEILV, where the HA can recognise sialic acid receptors and be activated by human host proteases, upon entry this fish virus would have to co-opt various mammalian host proteins for replication as well as restriction by mammalian interferon-stimulated genes, known restriction factors of IBV [9]. Thus, the zoonotic potential of such viruses (if any at all exists) remains unknown, as does the role of these viruses in IBV ecology. Nonetheless, characterisation of such IBV-like viruses is a pivotal step in understanding the diversity and evolutionary history of orthomyxoviruses, as well as their host range and ecology.

**Table 2:** Functional characteristics of the HA and NA glycoproteins from IBV-like viruses.

	HA				NA	
	Receptor Specificity ^1^	HA Activity ^2^	Fusogenic Activity ^3^	Protease Cleavage Profile	Enzymatic Activity ^6^	Inhibitor Susceptibility
IBV	α2,3-linked sialic acid α2,6 linked sialic acid	Yes	Fusogenic at a range of pH conditions (4.5–5.9)	Cleavable by various human TTSPs and KLKs ^5^	Yes	Peramivir > Zanamivir > Oseltamivir
SILV	Unknown, did not interact with sialic acids	No	Not detected ^4^	Not cleavable by human TTSPs and KLKs or trypsin	Not tested	Not tested
SAEILV	α2,3-linked sialic acid sialyated Lewis X	Yes	Fusogenic at a range of pH conditions (4.5–5.9)	Cleavable by TMPRSS4, TMPRSS13, KLK5, KLK14	Yes	Zanamivir > Peramivir > Oseltamivir
CSILV	α2,3-linked sialic acid	Yes	Not detected ^4^	Not cleavable by human TTSPs and KLKs or trypsin	Not tested	Not tested
WSEIV	monosialic ganglioside 2 (GM2)	No	Fusogenic at a range of pH conditions (4.5–5.9)	Not cleavable by human TTSPs and KLKs, cleavable by trypsin	Yes	Peramivir > Zanamivir > Oseltamivir

^1^ Determined by glycan microarrays; ^2^ Determined by hemagglutination assay using turkey and chicken RBCs; ^3^ Determines in a polykaryon assay; ^4^ Likely due to lack of cleavability; ^5^ Human type II transmembrane serine protease and human kallikrein protease; ^6^ Determined by an enzyme-linked lectin assay. Data from [56,57,58,60].

## 5. Conclusions and Future Directions

Through metagenomics and expanded surveillance for viruses in animals, it has become clear that a number of “human-restricted” viruses have far more complex ecologies that include animals, and IBV is a clear example of this. However, current evidence of IBV infection in animals is sporadic, and at present it is unclear whether animals are merely spillover hosts (i.e., reverse zoonosis) or if any may play a role as *bona fide* reservoirs for IBVs. There is mounting evidence for a putative role of pinnipeds in IBV ecology–serological detections from multiple populations, species and continents, and putatively sustained transmission. However, without dedicated long-term studies and the characterisation of further IBV genomes, the role of pinnipeds as IBV hosts may remain unclear. The identification of IBV-like viruses in amphibians and fish and the functional characterisation of their proteins provide insights into the evolutionary history of IBV. It is worth noting that the virus sequences (or protein products) identified from reverse zoonosis events, like the ones from pigs, have not been characterised in the same way those from IBV-like viruses have, which could provide novel insights into potential species adaptation. Further studies focused on virus isolation, relevant reverse genetics systems, and animal models, including fish species, would be invaluable for experimentally investigating host range, transmission dynamics, pathogenesis, and adaptation. That most of our evidence for IBV in animals is associated with reverse zoonosis highlights how unappreciated, yet common, this phenomenon is, further illustrating the extensive range of virus species shared between humans and animals.

Assuming the circulation of human or non-human IBV in animals, it is unclear (i) if these may pose a risk to humans or animals and (ii) how the sporadic introduction of human IBV into animal populations may influence the evolutionary ecology of these viruses in their animal hosts. Nonetheless, the identification of a diverse range of IBV-like viruses in fish and amphibians supports the notion of long-term co-evolution between IBVs and their animal hosts. This reinforces the view that these viruses have deep evolutionary roots within the animal kingdom, making it implausible to consider them as entities exclusive to humans.

## Figures and Tables

**Table 1:** Evidence for IBV infection in animals.

Animal (Species Where Available)	Location, Year	Detection Methods	Frequency of Animals Positive	Ref.
Animals in close contact with humans (pets, zoos, farms etc.)				
Horse	Japan, 1997	HAI	16/504 (3.2)	[13]
Horse	Canada, 1960–1963	Complement fixation assay	Numbers not reported (30)	[42]
Pigs	Japan, 1997	HAI	1/1030 (0.1)	[13]
Pigs	USA, 2010–2012	HAI, verified by NT	41/560 (7.3), 3 RT-PCR+ nasal swabs	[14]
Pigs	Great Britain, October 1991–February 1992	HAI, verified by NT and immunoblot	8/2000 (0.4)	[18]
Pigs	Taiwan, 2007–2017	HAI	31/15,983 (0.2), 3 nasal swabs with culturable IBV	[15]
Goats	Pakistan, 2023	Luminex assay to HA	32/452 (7.1)	[46]
Sheep	Pakistan, 2023	Luminex assay to HA	15/329 (4.6)	[46]
Dogs	Taiwan, 1971	Virus isolation from nasal swabs	1/372 (0.3)	[43]
Dogs	Japan, 2009–2020	NT, verified by immunoblot	6/366 (1.6)	[17]
Guinea pigs	Ecuador	ELISA with whole virus, recombinant HA and NP, verified by immunoblot	28/40 (70)	[44]
Bamboo rats	China, 2020	Metagenomics	1 full genome recovered	[45]
Chimpanzees	Netherlands, 1986, 1992, 1998, 2000	Magnetic bead-based assay, verified by immunoblot	80/305 (26.2)	[16]
Gorillas	Not specified, reported in 2014	Magnetic bead-based assay, verified by immunoblot	45/77 (58.4)	[16]
Orangutans	Indonesia, 1994–1998	Magnetic bead-based assay, verified by immunoblot	135/179 (75.4)	[16]
Wild animals				
Harbour seals (*Phoca viulina*) and grey seals (*Halichoreus grypus*)	Netherlands, 1995–1999	HAI and ELISA to HA, NA, and NP	0/580 < 1995 8/391 (2) 1995–1999 1 RT-PCR+ throat swab in 1999	[20]
Harbour seals (*Phoca viulina*) and grey seals (*Halichoreus grypus*)	Netherlands, 2002–2012	HAI	10/71 2010–2011 0/454 (0) all other years	[19]
Caspian seals (*Phoca capsica*)	Caspian Sea, 1997–2000	ELISA with whole virus	5/77 (6)	[22]
South American fur seals (*Arctocephalus australis*)	Uruguay, 2004	HAI	25/37 (67.6)	[47]
Caspian seals (*Phoca capsica*)	Caspian Sea, 2007–2017	HAI	14/70 (20)	[23]
Caspian seals (*Phoca capsica*)	Caspian Sea, 2020	metagenomics, confirmed by RT-PCR	partial PB2 and NS2 gene segment detected in spleen of dead animal in 2020	[24]
Hooded seal (*Cystophora cristata*) Grey seal (*Halichoerus grypus*) Harbour seal (*Phoca vitulina*) Harp seal (*Pagophilus groenlandica*)	Canada, 1994, 2005	ELISA, followed up by HAI	29/394 (7.4%) by ELISA all negative by HAI	[21]

## Data Availability

No new data were created or analysed in this study.
